# The Divide–Survive Matrix: decoupling proliferative drive from intrinsic survival architecture in oncology

**DOI:** 10.3389/fonc.2026.1821478

**Published:** 2026-07-24

**Authors:** Glen Macdonald Heavenor

**Affiliations:** Independent Researcher, Glasgow, United Kingdom

**Keywords:** biologically aligned prognosis, Divide-Survive Matrix framework, intrinsic survival architecture, proliferative drive, tumor aggression assessment

## Abstract

Tumor behavior is commonly interpreted through proliferative indices and histopathological classification. However, clinical aggression and therapeutic persistence are not fully explained by proliferation alone. Lesions with comparable proliferative activity may demonstrate markedly different trajectories of progression, resistance, or metastatic potential. These discrepancies suggest that proliferative drive and baseline survival programs represent biologically distinct dimensions of tumor behavior. We introduce the Divide–Survive (DvS) Matrix as a structured two-dimensional representation integrating proliferative drive—commonly approximated by Ki-67—and intrinsic survival architecture (ISA), reflecting biological programs that support persistence independent of proliferation. Proliferative positioning may be stratified according to institutional practice or multidisciplinary consensus, including banded aggression categories, ensuring that tumor placement remains clinician-determined rather than prescriptive. ISA is conceptually distinct from therapy-induced adaptive phenomena such as metastatic drug endurance and represents baseline biological positioning at the time of diagnostic biopsy. Matrix positioning is intended as a qualitative-to-semi-quantitative interpretive exercise, guided by multidisciplinary clinical judgement rather than algorithmic assignment. By formally decoupling proliferative activity from survival architecture, the matrix offers a visual interpretive structure through which multidisciplinary teams may assess tumor aggression, clarify discordant behavior, and refine therapeutic reasoning. If prospectively evaluated, this approach may support more biologically aligned prognostic interpretation and therapeutic contextualization across histological subtypes.

## 1 Introduction

In clinical practice, tumors with comparable proliferative indices may follow markedly different trajectories of progression, response, and persistence. Such discordance indicates that proliferation alone does not fully account for tumor aggression or therapeutic outcome (1, 2). The Divide–Survive (DvS) Matrix addresses this gap. We hypothesize that decoupling proliferative drive from intrinsic survival architecture provides a potentially more biologically coherent interpretive framework for interpreting tumor aggression than proliferation metrics alone.

Histopathological classification and grading systems remain foundational to oncology (3). Yet biological programs that enable persistence—independent of expansion rate—are not always made explicit within existing interpretive structures. Relapse following apparent response, sustained progression despite modest proliferation, and metastatic endurance often reflect mechanisms distinct from proliferative velocity (4).

**Figure 1:**
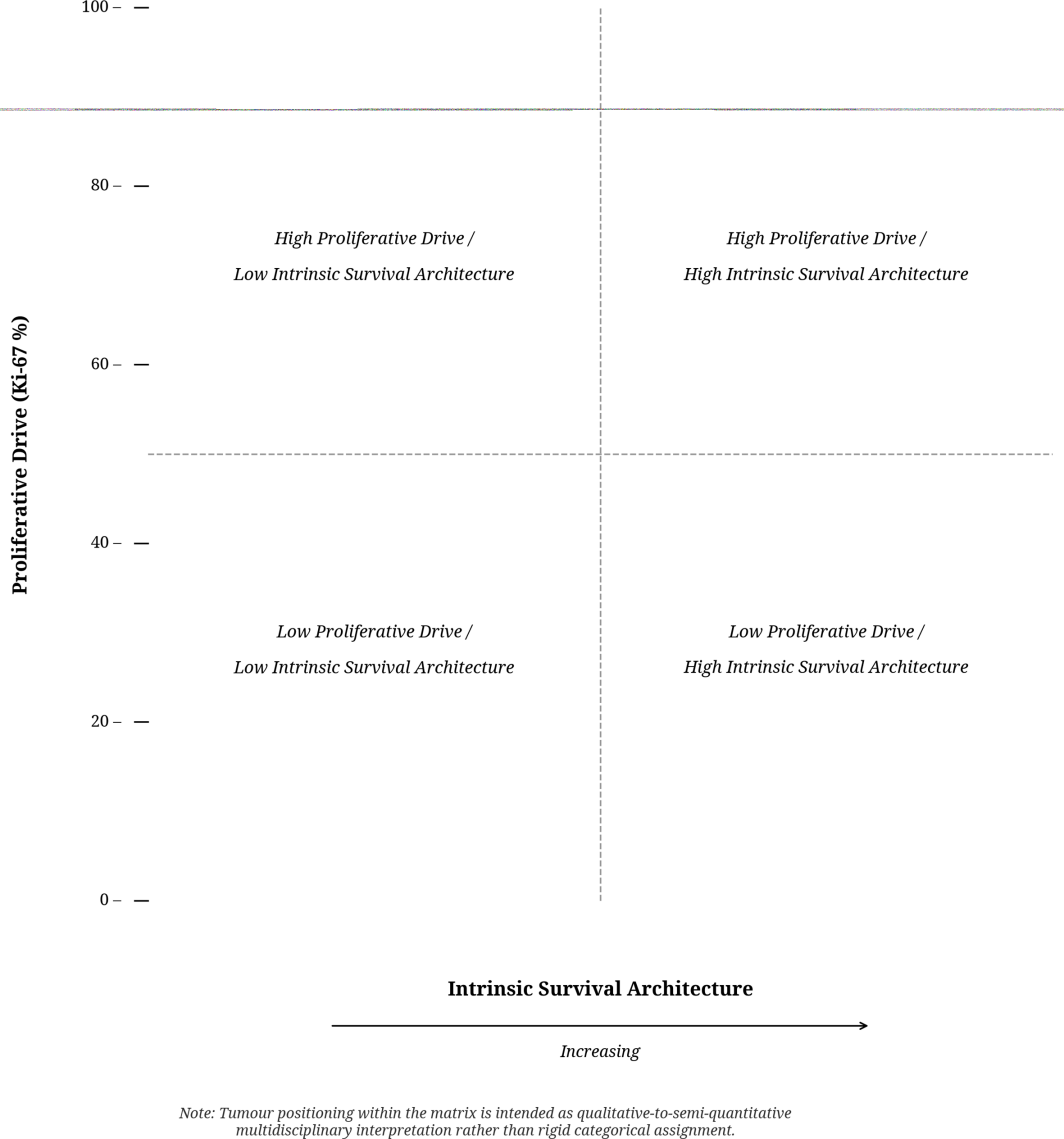
The Divide–Survive (DvS) Matrix. A two-dimensional representation of tumor behavior across proliferative drive (vertical axis) and intrinsic survival architecture (horizontal axis). Proliferative drive reflects measurable expansion pressure, commonly approximated by Ki-67 or institutional stratification. Intrinsic survival architecture represents baseline biological programs supporting persistence independent of proliferation. Positioning is determined through multidisciplinary clinical interpretation at the time of diagnostic biopsy. Tumor positioning within the matrix is intended as a qualitative-to-semi-quantitative interpretive exercise rather than rigid categorical assignment.

The Divide–Survive Matrix addresses this gap by organizing tumor behavior within a two-dimensional structure defined by proliferative drive and intrinsic survival architecture. Proliferative drive reflects measurable expansion pressure. Intrinsic Survival Architecture (ISA) refers to the integrated biological programs that support tumor persistence independent of proliferative velocity. These include both tumor-cell intrinsic properties—such as lineage plasticity (5), stem-like states (6), metabolic adaptability (7), and apoptotic resistance—and context-dependent survival supports, including stromal integration (8), immune evasion, and niche dependence (9, 10). It is important to note that molecular pathway signaling, including major oncogenic signaling pathways, may contribute to both proliferative drive and survival architecture simultaneously; the DvS framework does not require mechanistic exclusivity between axes, only that their functional outputs are separable and independently interpretable at the clinical level. The conceptual structure of the framework is represented in **Figure 1**.

### 1.1 Relationship to existing conceptual models

The DvS Matrix does not replace existing conceptual frameworks but seeks to integrate biologically relevant dimensions of persistence and proliferation into a single interpretive structure. Proliferation-based models and grading systems primarily describe expansion velocity but do not explicitly separate persistence architecture from proliferative drive. Dormancy frameworks explain low-divide persistence but do not integrate highly proliferative, survival-dominant states within the same coordinate system. Stemness and plasticity models focus on differentiation state (11, 12) and cellular identity but do not structurally decouple proliferative pressure from persistence architecture. Evolutionary fitness models address adaptation and clonal selection but are less directly interpretable at the time of diagnostic biopsy.

By formally separating these axes, the DvS Matrix provides a coordinate system that generates distinct interpretive logic for tumors that might otherwise be grouped together under conventional grading or classification. In this respect, the framework operationalizes biological distinctions that are often implicit in clinical experience but are not yet made structurally explicit in routine multidisciplinary discussion (13, 14).

Used alongside established grading systems, this structure offers an additional lens through which multidisciplinary teams may place tumors at the time of diagnostic biopsy and interpret discordant clinical behavior.

## 2 Differentiation and survival architecture

Absence of terminal lineage markers may arise through two distinct biological routes: arrested differentiation (never acquired) or dedifferentiation (lost). Although both converge phenotypically as marker-poor states, their developmental trajectories differ.

Within the matrix, these states inform positioning along the survival axis rather than directly determining proliferative drive. Arrested differentiation may reflect incomplete lineage commitment with retained adaptability, whereas dedifferentiation represents regression from a previously mature state. In both settings, reduced lineage fixation may facilitate persistence through metabolic reprogramming, cellular flexibility, or microenvironmental adaptation (15–17).

Marker-poor status does not inherently equate to high proliferative activity. A tumor may exhibit limited proliferative drive while maintaining robust survival architecture, while highly proliferative tumors may retain strong lineage identity yet remain biologically vulnerable. Differentiation status therefore informs survival positioning but does not independently define tumor aggression.

### 2.1 Operationalizing the intrinsic survival architecture axis

To facilitate multidisciplinary interpretation, the ISA axis may be evaluated using a qualitative-to-semi-quantitative approach based on integrated biological and architectural features. The following table provides interpretive guidance; it is not intended as a rigid deterministic scoring system, and marker selection should remain context-specific and subject to multidisciplinary judgement (**Table 1**).

**Table 1:** Interpretive guidance for Intrinsic Survival Architecture (ISA) axis positioning within the DvS Matrix.

ISA positioning	Biological characteristics	Indicative markers (examples)	Clinical implication
Low ISA	High reliance on proliferative drive; limited pathway redundancy; high apoptotic sensitivity.	Low or absent functional survival signaling (e.g., pS6−, p-AKT−); mature lineage markers present.	Potentially vulnerable to standard cytotoxic therapies targeting the divide axis.
Intermediate ISA	Emerging plasticity; partial microenvironmental dependence; mixed apoptotic resistance.	Heterogeneous or weak functional survival signaling; partial loss of mature lineage markers.	May require sequential or combined modalities; risk of adaptive resistance.
High ISA	Pathway-dependent survival dominance; high apoptotic resistance; metabolic adaptability; stem-like or marker-poor state.	Strong, diffuse functional survival signaling (e.g., pS6+, p-AKT+); marker-poor or dedifferentiated phenotype.	Resistant to standard cytotoxics; warrants consideration of direct survival pathway targeting.

Positioning is determined through multidisciplinary clinical interpretation at the time of diagnostic biopsy.

### 2.2 Illustrative positioning within the DvS Matrix

The following example illustrates how two tumors with identical proliferative indices may occupy distinct positions within the DvS Matrix and thereby warrant different interpretive approaches.

Consider two tumors presenting with an identical, moderate proliferative index (Ki-67 of approximately 15%). Under a proliferation-only interpretive framework, both might be managed similarly. Tumor A exhibits strong, mature lineage markers and absent functional survival signaling. It is positioned as Moderate Divide/Low ISA. Its persistence relies principally on its moderate expansion rate, and it may remain vulnerable to interventions that interrupt the cell cycle. Tumor B is marker-poor, exhibits stem-like features, and demonstrates strong, diffuse functional survival signaling. It is positioned as Moderate Divide/High ISA. Its persistence is driven by a robust, pathway-dependent survival architecture rather than by rapid expansion. The DvS Matrix makes this distinction visible at the time of biopsy, enabling the multidisciplinary team to consider whether the therapeutic strategy should be directed primarily at the divide axis, the survival axis, or both.

This example is not intended to prescribe a specific therapeutic algorithm but to illustrate the interpretive value of separating proliferative drive from survival architecture as distinct, independently assessable dimensions of tumor behavior.

## 3 Discussion

The Divide–Survive (DvS) Matrix proposes a structured decoupling of proliferative drive and intrinsic survival architecture as complementary dimensions of tumor aggression. While proliferation indices such as Ki-67 remain central to histopathological assessment, clinical experience and biological research consistently demonstrate that expansion rate alone does not fully explain persistence, relapse, or metastatic endurance. By separating proliferative pressure from baseline survival programming, the framework aims to clarify discordant clinical behavior without displacing established grading systems.

Importantly, the DvS Matrix is positioned at the time of diagnostic biopsy. It does not depend upon treatment exposure or longitudinal outcome data, nor does it introduce new biomarkers. Rather, it organizes already recognized biological phenomena—plasticity, stem-like states, metabolic adaptability, and microenvironmental integration—into a two-dimensional interpretive structure. In this respect, it reframes known biology rather than proposing a novel molecular pathway.

**Figure 2:**
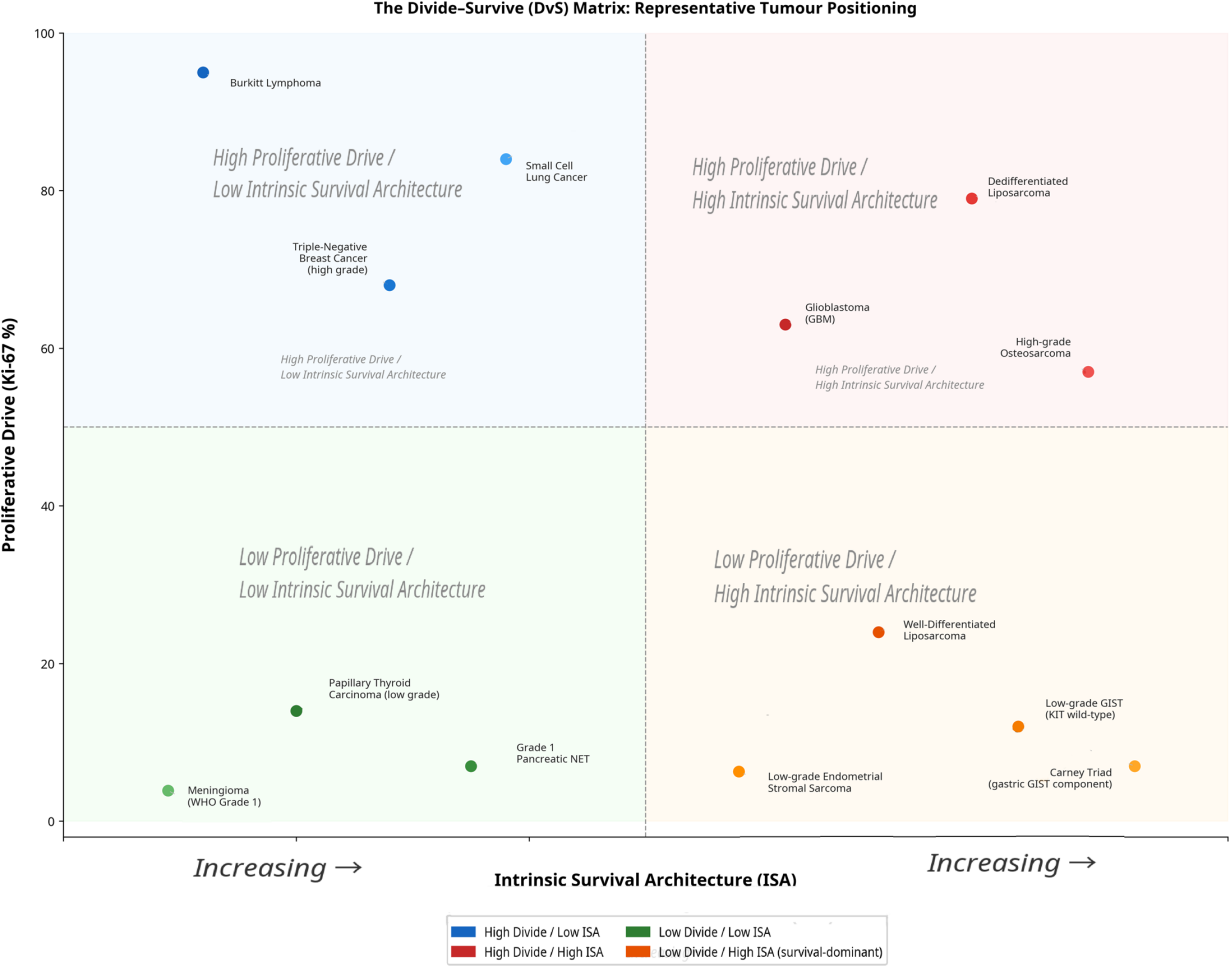
The Divide–Survive (DvS) Matrix: Representative Tumor Positioning.

**Figure 3:**
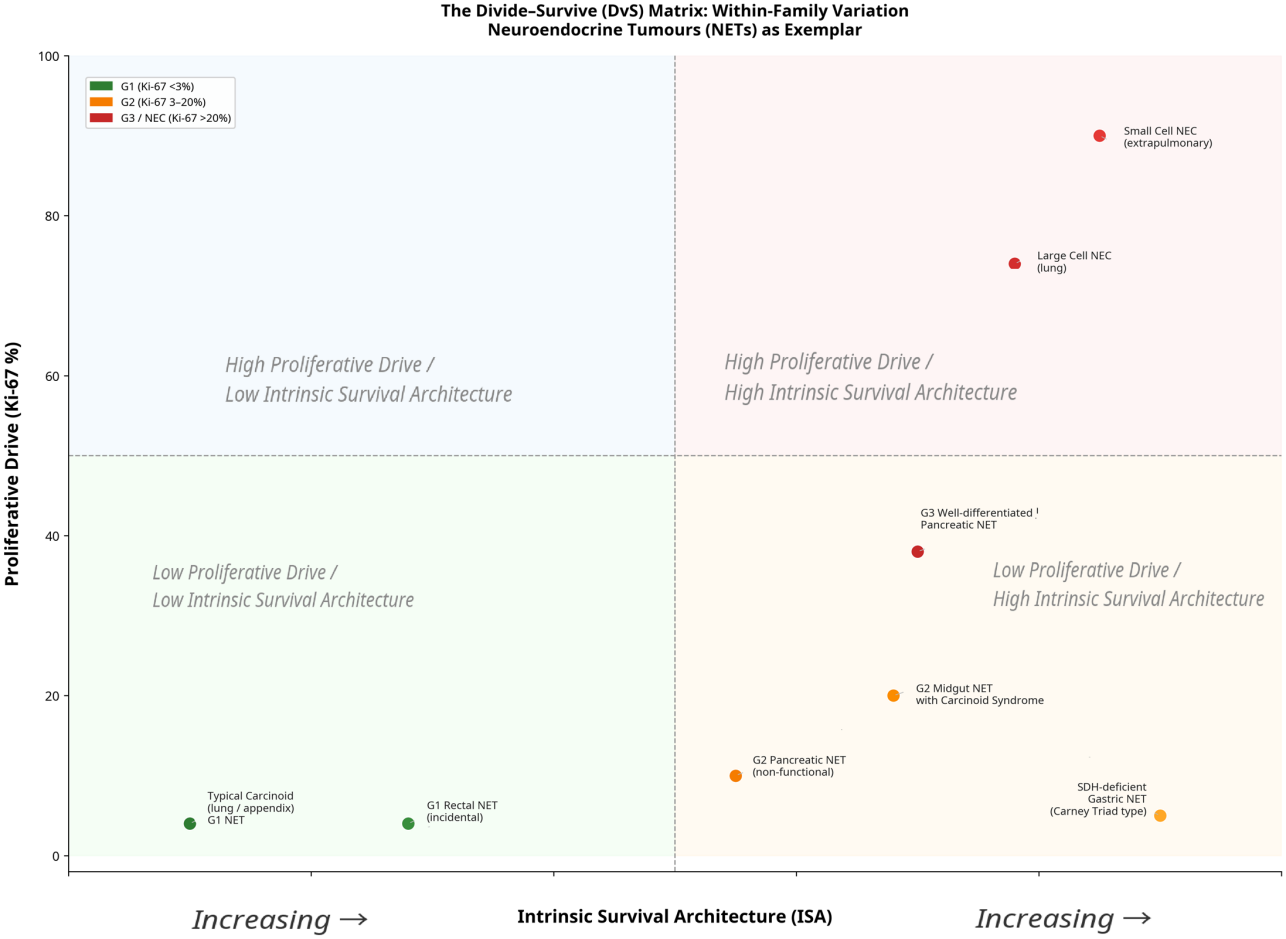
The Divide–Survive (DvS) Matrix: Within-Family Variation—Neuroendocrine Tumors (NETs) as Exemplar.

The biaxial model may also help articulate biological tensions that are sometimes implicit within conventional classification systems. Tumors categorized within similar grades may occupy distinct positions when proliferative velocity and intrinsic survival architecture are considered separately. Such differentiation does not invalidate existing taxonomies; instead, it provides an additional lens through which multidisciplinary teams may interpret variability in progression or response. Representative tumor positioning within the DvS Matrix is illustrated in **Figure 2**. with within-family variation further exemplified in **Figure 3**. (The co-ordinates represent illustrative conceptual placements within the DvS framework, rather than evidence-based quantitative estimates or actual measured KI-67 values or validated numerical ISA scores). The framework therefore operates as a complement rather than a substitute.

Importantly, the DvS framework does not require advanced molecular profiling to permit clinically meaningful interpretive positioning. Many components contributing to Intrinsic Survival Architecture (ISA)—including differentiation state, architectural disruption, lineage instability, necrosis, and broader indicators of biological persistence—are already evaluated, either explicitly or implicitly, within routine histopathological and multidisciplinary assessment. In this respect, the DvS Matrix may be viewed not as a replacement for existing grading practices, but as a structured visual-cognitive framework through which these biological dimensions can be spatially contextualized alongside proliferative drive.

Beyond conceptual framing, this perspective may encourage greater structural interrogation of diagnostic data at the time of biopsy. Immunophenotypic profiles and lineage features that are often collapsed into residual or “not otherwise specified” categories may, when viewed through the lens of proliferative drive and intrinsic survival architecture, prompt more deliberate reflection on how differentiation state and persistence biology are represented within a given tumor. In this sense, the principal contribution of the framework is cognitive rather than algorithmic.

### 3.1 Pathway to validation

While the DvS Matrix is currently proposed as a qualitative-to-semi-quantitative interpretive framework, it provides a clear and testable pathway for future validation. Retrospective studies could examine existing clinicopathological cohorts to determine whether tumors with comparable proliferative indices but differing ISA profiles—as assessed through functional immunophenotypic and pathway-associated features—demonstrate divergent patterns of persistence, recurrence, metastatic behavior, or therapeutic response (18–20). Such analyses would not require new tissue collection and could be performed across archived diagnostic material. Prospective operationalization could then refine the specific marker panels required to standardize ISA axis positioning across different histological subtypes, with the goal of establishing reproducible interpretive criteria for multidisciplinary use.

### 3.2 Limitations

Several limitations must be acknowledged. The DvS Matrix is a hypothesis-generating structure and has not yet been prospectively validated across tumor types. Its axes represent conceptual dimensions derived from established biological principles (21) rather than quantitatively calibrated metrics. The matrix does not replace established grading systems, staging criteria, or histopathological interpretation (22). Rather, it is intended for immediate comparative use alongside existing multidisciplinary conclusions regarding tumor aggression. It introduces no novel biomarkers, instead organizing recognized biological dimensions into a structured representation. Future work may explore operationalization, reproducibility across disease contexts, and potential integration with molecular profiling platforms. However, such validation efforts should not obscure the central proposition: that tumor aggression may be more coherently interpreted when proliferative expansion and intrinsic survival programming are considered as related but distinct variables.

Within this framework, three practical questions may guide interpretive reflection at the time of biopsy:

1. Where does the tumor reside within the proliferative drive–survival architecture matrix?
2. How does this position compare with the biological profile of tumors traditionally grouped within the same diagnostic category?
3. What implications might this relative positioning have for expected behavior and therapeutic vulnerability?

Intrinsic survival architecture currently relies on composite clinical interpretation informed by established biological knowledge. While future work may refine quantitative measures for this axis, matrix positioning does not depend on new assays to be operationally useful within MDT discussion.

Placement reflects tumor biology at the time of diagnostic biopsy. Dynamic changes under therapeutic pressure may shift positioning and warrant reassessment.

### 3.3 Conclusion

In summary, the DvS Matrix offers a biologically grounded structured visual-cognitive framework that decouples proliferative drive from intrinsic survival architecture. By making explicit a distinction that is often implicit in tumor biology, it seeks to refine—not replace—existing diagnostic and grading paradigms.

The framework is positioned as a complementary interpretive structure for use alongside established histopathological and molecular assessment. Its principal value lies in providing multidisciplinary teams with a structured means of articulating biological discordance—particularly in cases where proliferative indices do not adequately explain clinical behavior. Future prospective validation will be essential to determine the reproducibility and clinical utility of ISA axis positioning across tumor types and treatment contexts.

## Data Availability

No original data were generated or analyzed for this study. This article presents a hypothesis-generating conceptual framework based on published literature.
